# Shift in the isoelectric-point of milk proteins as a consequence of adaptive divergence between the milks of mammalian species

**DOI:** 10.1186/1745-6150-6-40

**Published:** 2011-07-29

**Authors:** Nora Khaldi, Denis C Shields

**Affiliations:** 1 UCD Conway Institute of Biomolecular and Biomedical Research, School of Medicine and Medical Sciences, University College Dublin, Dublin 4, Republic of Ireland; 2 UCD Complex and Adaptive Systems Laboratory, University College Dublin, Dublin 4, Republic of Ireland

## Abstract

**Background:**

Milk proteins are required to proceed through a variety of conditions of radically varying *pH*, which are not identical across mammalian digestive systems. We wished to investigate if the shifts in these requirements have resulted in marked changes in the isoelectric point and charge of milk proteins during evolution.

**Results:**

We investigated nine major milk proteins in 13 mammals. In comparison with a group of orthologous non-milk proteins, we found that 3 proteins κ-casein, lactadherin, and muc1 have undergone the highest change in isoelectric point during evolution. The pattern of non-synonymous substitutions indicate that selection has played a role in the isoelectric point shift, since residues that show significant evidence of positive selection are much more likely to be charged (p = 0.03 for κ-casein; p < 10^-8 ^for muc1). However, this selection does not appear to be solely due to adaptation to the diversity of mammalian digestive systems, since striking changes are seen among species that resemble each other in terms of their digestion.

**Conclusion:**

The changes in charge are most likely due to changes of other protein functions, rather than an adaptation to the different mammalian digestive systems. These functions may include differences in bioactive peptide releases in the gut between different mammals, which are known to be a major contributing factor in the functional and nutritional value of mammalian milk. This raises the question of whether bovine milk is optimal in terms of particular protein functions, for human nutrition and possibly disease resistance.

This article was reviewed by Fyodor Kondrashov, David Liberles (nominated by David Ardell), and Christophe Lefevre (nominated by Mark Ragan).

## Background

The isoelectric point (*pI*) and charge of a protein is important for solubility, subcellular localization, and interaction. There is a correlation between subcellular location and protein *pI *[1,2]. Proteins in the cytoplasm possess an acidic *pI *(*pI *< 7.4), while those in the nucleus have a more neutral *pI *(7.4 <*pI *< 8.1) [1,2]. It has also been shown that the *pI *can vary greatly, depending on both insertion and deletions between orthologs, and the ecology of the organism [3]. Kirga et al [3] have shown that the *pI *of membrane proteins of bacteria correlates with their ecological niche, and changes dramatically from acidic to basic. For example, some prokaryotes that infect human have a *pI *that reflects their localization in the human body, compensating for the *pH *change. *E. coli *that resides in the intestines has more acidic proteins, and *H. pylori *that infects the acidic stomach has more negatively charged proteins [3].

For highly abundant proteins, shifts in their *pI *can impact on the function of organs that interact with them. Purtell et al [4] examined the effects of change in isoelectric point (*pI*) on renal handling of albumin molecules. The authors showed that the increase of the *pI *caused an increase in heterologous albumin secretion and increased nephron permeability.

Milk proteins travel through the various mammalian digestive systems with their different compartments and *pH *levels. For example, carnivorous species possess very acidic stomachs compared to herbivores, and orthologous milk proteins need to travel and perform their function in all these systems. Because of these differences we might expect to observe adaptation of the milk proteins in order to perform orthologous functions, or an adaptation of the *pI *to a new acquired functionality. Large differences in the *pI *of milk proteins might have important consequences on the structure, properties, functionality and interaction of these proteins.

In this work we investigate the evolutionary changes in the *pI *values of the milk proteins (Table 1) as a one-dimensional indicator of critical shifts between orthologous milk proteins that might reflect responses to environmental and functional changes between the different mammalian species.

**Table 1:** Function of milk proteins

Protein	Role	Milk fraction
α-S1-casein, β-caseinm and κ-casein	~80% of bovine and 20-45% of human milk protein. Phosphoprotein carriers of minerals and trace elements.	Casein micelles
α-lactalbumin	Calcium and other carrier, lactose synthesis [26]	Whey
Lactoferrin	Iron and other metal binding [27], antimicrobial, antiviral [28], antioxidative, cell growth regulator	Whey
Lactadherin	Also known as Milk Fat globule factor 8 (Mfge8); bactericidal and apoptotic properties [29].	Milk fat globule; digestion resistant
Mucin 1	Modulates bacterial adhesion [29]	Milk fat globule; digestion resistant
Xanthine oxidase/dehydrogenase	Fat globule secretion [30] Innate immunity/oxidation [31]	Milk fat globule
Butyrophilin	~40% of protein in Milk Fat Globule Membrane; fat globule secretion [29]	Milk fat globule; rapidly degraded

We show that the shifts do not simply reflect differences in sequence lengths between the milk orthologous proteins, and are likely driven by selection. Both sequence length and selection have been recently shown to explain the observed differences in *pI *between mammalian orthologs [5]. We argue that the differences in the digestive systems due to *pH *and compartmentalization of the different mammals is not the sole driver of major changes in *pI*, and that these selective changes might be due to functional divergence of the protein.

## Results and discussion

### *Calculation of pI*

**Figure 1:**
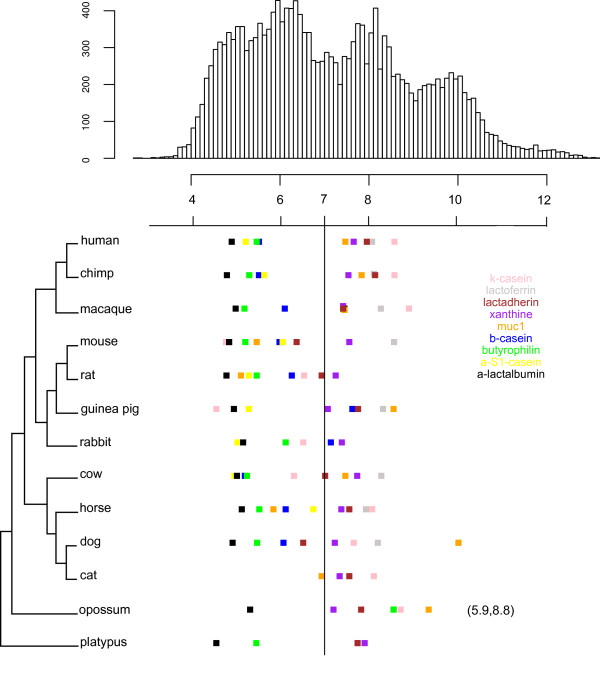
***pI *values for the nine major milk proteins in 13 mammalian species compared to the *pI *of the human proteome**. The top histogram represents the *pI *distribution of the human proteome. The histogram's x-axis is shared with that of the major milk proteins' *pI *shown below. The Colors indicate the different milk proteins. The tree on the left is the mammalian species tree from Benton and Donoghue [23]. Two extra *pI *values are represented between brackets at the opossum level, these represent the *pI *of the reported extra copies of κ-casein this species possess [6]. The values are for the proteins with the accession numbers FJ548612 and FJ548626 respectively.

To investigate if the milk proteins have experienced shifts in their *pI *between different mammalian species, we selected nine milk proteins that share three main conditions; firstly they are representative of one of the three components of milk (casein, whey, milk-fat-globules); secondly they are present in at least eight mammalian species allowing for comparative genomics; finally the proteins possess a well characterized protein and cDNA sequence. We calculated the *pI *of the milk proteins after removing defined signal peptides. Some proteins show quite strong evolutionary conservation of *pI *(Figure 1). α-S1-casein, β-casein, α-lactalbumin, and butyrophilin subfamily 1 member A1 have only changed slightly between species and remain acidic through the tree (Figure 1). Similarly, xanthine dehydrogenase/oxidase is maintained in the neutral range in all mammals (Figure 1).

**Figure 2:**
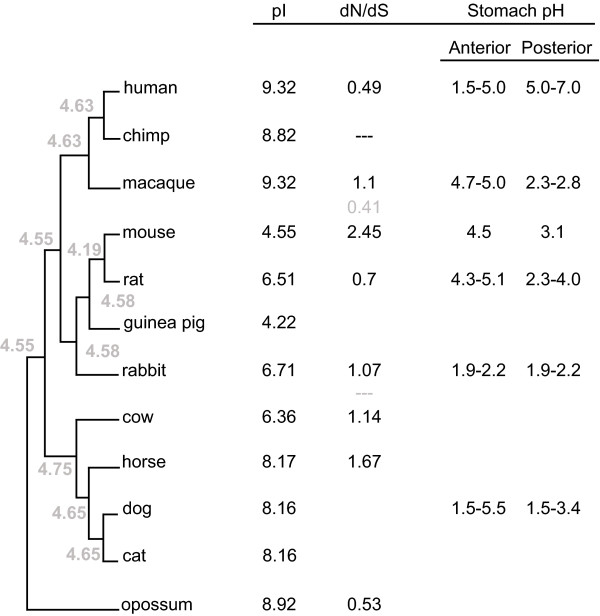
**Ancestral reconstruction of κ-casein and the representation of *pI, dN/dS *ratio and the stomach *pH *values**. Ancestral values are represented in grey. The ratio *dN/dS *was calculated only for species with a well-defined cDNA (this is not the case for guinea pig, cat, and dog). When the ratio *dN/dS *is undefined due to extremely small *dS *values we used the symbol "--". Despite the fact that the *pH *of other digestive compartments can show marked differences between mammals, we chose only to represent the stomach *pH *values, as this compartment is the main first barrier for milk proteins. A review of the *pH *values is reported in Table 5 of the following reference [24] (p366), we could not find well-defined values for chimp, horse, cow, and guinea pig.

However, some proteins show more dramatic changes in one or multiple branches on the tree. Thus, κ-casein *pI *has apparently, under a parsimonious model, shifted from a basic ancestor to an acidic *pI *on the branch prior to the speciation of rodents, guinea pig, and rabbit (mouse *pI *= 4.75, rat *pI *= 6.53, guinea pig *pI *= 4.53, and rabbit *pI *= 6.51). Nevertheless, rat and rabbit are substantially less acidic than mouse and guinea pig, suggesting that more than one change in constraint on κ-casein *pI *during evolution in these lineages. κ-casein in cow has a much lower *pI *than horse, again suggesting an independent shift in constraint. Indeed, the most parsimonious scenario accounting for the current κ-casein *pI *values represented in Figure 1 and Figure 2 will require two changes in the ancestors of mouse and cow from an ancestral basic *pI *value to a more acidic observed value in both these species. In contradiction to this result, an ancestral reconstruction shows that the ancestor of κ-casein carried an acidic *pI*, and that further on in evolution this value shifted in a multitude of species to the current observed basic values (Figure 2 shows at least four independent shifts: in macaque, the ancestor of human and chimp, horse, and the ancestor of dog and cat). Besides according to this reconstruction all the current *pI *values are higher than the ancestral values (Figure 2), including the *pI *values of mouse and rat κ-casein (Figure 2). However it is known that ancestral reconstruction is somewhat unreliable especially at sites with alignment gaps. We thus cannot argue for such a scenario, and from the current value a parsimonious scenario with fewer events is more likely to explain the current *pI *values in the κ-casein orthologs (Figure 1 and Figure 2).

It has been shown that platypus contains two extra copies of κ-casein [6]. These two copies have very different *pI *values ranging from acidic to basic, with *pI *= 5.9 for FJ548612, to *pI *= 8.8 for FJ548626 (Figure 1). Contrary to the other observed shifts in *pI *represented in Figure 1, the great shift in *pI *between the κ-casein copies cannot be explained by interspecies differences. It is noticeable that the pI of the current κ-casein orthologs is much higher than that of the ancestor values (Figure 2). However mouse and guinea pig seem to be an exception to this observation. It is unclear how much this is due to real *pI *shifts or to and artifact of the method of *pI *calculation.

Lactadherin has shifted at least twice on the tree. It is basic in the two outgroup species opossum (*pI *= 7.83) and platypus (*pI *= 7.75), in primates (*pI *= 7.96 human, *pI *= 8.17 chimp, *pI *= 7.42 macaque), and in guinea pig (*pI *= 7.76), but seems to have shifted independently twice to acidic/neutral, once in rodents (*pI *= 6.36 in mouse, and *pI *= 6.9 in rat), and another time in dog (*pI *= 6.45 in dog).

The pattern of *pI *change of muc1 protein shows a number of potential changes, shifting in two independent lineages to a lower *pI *in both rodents (mouse *pI *= 5.45, and rat *pI *= 5.09) and horse (*pI *= 5.83).

### Are the shifts in the *pI* of some milk proteins  important compared to whole proteome comparison?

What appear as dramatic changes between the *pI*s of κ-casein, lactadherin, and muc1 orthologs, might not seem so dramatic compared to the changes across the entire proteome for non-milk protein orthologs.

To investigate this, we considered all the orthologous proteins in the 13 mammals (human, chimp, monkey, mouse, rat, guinea pig, rabbit, cow, horse, dog, cat, opossum, platypus). We considered a shift in *pI *between human and mouse to be high if it was greater than 0.92, and an identical value between human and cow (Additional File 1; see Methods for the rationale behind the choice of these cut-offs). We further tested for the significance of this threshold by randomly assigning *pI *values to proteins, and found that our set thresholds are in all cases significant (p < = 0.01).

**Figure 3:**
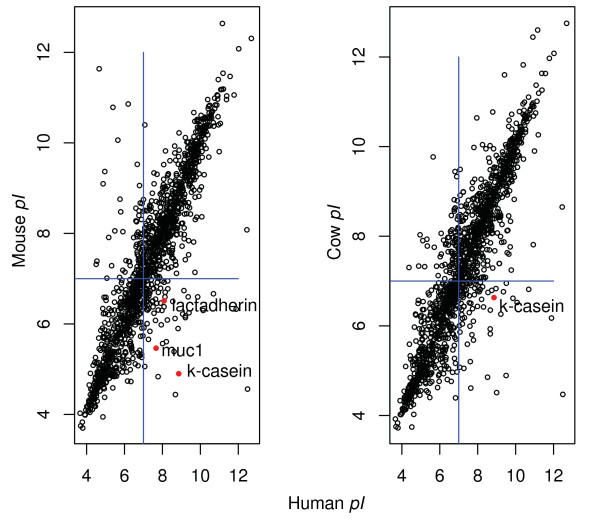
***pI *values of all the orthologous proteins in human, mouse, and cow**. The vertical and horizontal lines represents the neutral *pH *= 7. Most proteins have a similar *pI *between species, with some exceptions lying out on both sides of the diagonal. The red dots represent the three milk proteins that have the highest shift (κ-casein, lactadherin, and muc1).

Figure 3 shows that κ-casein, lactadherin, and muc1 stand out on the figure as being part of a very small proportion of proteins that have shifted dramatically in *pI*, from being basic in man to being acidic in mouse (κ-casein, and lactadherin), from being basic/neutral in human to being acidic in mouse (muc1), or from being neutral/acidic in cow to being basic in man (κ-casein). Figure 3 also shows that most proteins conserve their *pI *despite the evolutionary distances separating human, mouse, and cow. These large shifts seen for certain milk proteins are therefore unexpected for typical proteins that have conserved their function in evolution.

### *Differences in length between orthologs due to insertions or deletions are associated with the pIshift in certain proteins*

The change in *pI *between the milk proteins may reflect amino acid replacement at a number of residues, or they might be due to large insertions or deletions that cause large changes in *pI*. This has been shown to be the major reason behind the shift in *pI *between mammal proteins carried out by Alendé and co-authors [5]. For κ-casein, the shifts do not appear to relate to size differences, since the sequence length between human and mouse is very similar, and the extra amino acid in human does not account for the difference (Table 2). However, we observe noticeable changes in length between lactadherin and muc1. For lactadherin, human is 76 residues shorter than mouse (Table 2). When the regions in mouse that are not aligned with those found in human are removed, the *pI *is 7.7, close to that of human *(pI *= 8.0). For muc1, the human protein is much longer than the mouse sequence. However, the *pI *of the human regions alignable with mouse muc1 was 7.12, broadly similar to the *pI *of the overall protein (7.47).

**Table 2:** Sequence lengths for eight milk proteins in human, mouse, and cow

	Muc1	lactadherin	κ-casein	β-casein	α-casein	butyrophilin subfamily 1 member A1	xanthine dehydrogenase/oxidase	lactoferrin
Human	1255	387	182	226	185	526	1333	710
Mouse	630	463	181	231	313	524	1335	707
Cow	580	427	190	224	214	526	1332	708

These results show that for Lactadherin the change in *pI *is mainly due to the mouse insertion. However this scenario does not account for the change in *pI *for muc1 and κ-casein where both shifts are accounted for by amino acid replacements between human and mouse.

### *Selection causing pIchange*

**Figure 4:**
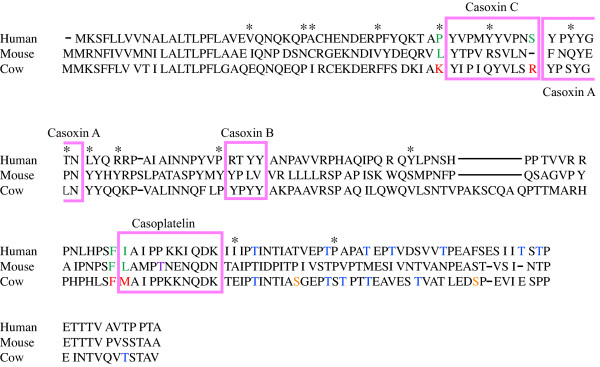
**Alignment of κ-casein between human, mouse, and cow**. The sequences of casoxin peptides A, B, and C are in the pink colored boxes. Cleavage sites are to the right of the red residues, while green residues are the corresponding residues that are not cleavable by the same enzyme in human and mouse. Casoxin-A and C are cleaved by a pepsin-trypsin digest for the former, and a trypsin digest for the later [12]. The peptide Casoplatelin [25] that inhibits ADP-induced platelet aggregation and fibrinogen binding is also represented on the figure together with the chymosin/rennin cleavage site between the F and M residues in red (while the same positions are in green in human and mouse). Horizontal lines represent gaps. Stars indicate sites that were predicted to be under positive selection (see results). Orange residues have been shown in the literature to undergo phosphorylation. Blue residues have been shown in the literature to undergo glycosylation. One potential phosphorylation site indicated in lavender in mouse.

Can selection have contributed to the change in *pI*? A recent study of the *pI *of mammalian proteins argues that selection has contributed to some of the *pI *shifts between orthologous proteins [5]. We searched for evidence of positive selection using the Sitewise Likelihood Ratio (SLR) method for the estimation of selection [7] in each site of the alignment of human, mouse, and cow for muc1 and κ-casein. SLR is a direct test of whether a particular site is evolving in a non-neutral fashion, inspecting the excess of non-synonymous over synonymous DNA changes; and indicates which sites in the protein have strong evidence of positive selection, which correspond to sites that are unusually variable. For κ-casein we found evidence of 14 sites presenting positive selection (p < = 0.043; Figure 4). Eleven of these sites change the *pI *of the protein, and 7 of those also change the overall charge of the protein at neutral *pH*. Only four positively selected sites have not affected the *pI *of the protein, and are not known to be implicated in any side modifications of the protein. We find that there are significantly more sites that affect the *pI *that have undergone positive selection compared to all other sites that do not affect the *pI*. Thus, there are significantly more sites undergoing positive selection and that have an impact of the net charge of the protein compared to all other neutral sites (p = 0.03; 22% for charged residues versus 5% for neutral sites). Under a random distribution of the positively selected sites detected in the human κ-casein protein sequence, we will expect an average of 8.4% sites that undergo positive selection whether these are charged or neutral, which is less than the observed 22% charged sites that have undergone positive selection.

Given that so many residues are experiencing adaptation in the human κ-casein and have a direct impact on the *pI *argues for adaptive changes in the *pI *of κ-casein. To further examine if positive selection has played a role in the evolution of κ-casein, we calculate the ratio of the rate of non-synonymous over synonymous substitutions (*dN/dS*). Figure 2 shows that the mouse κ-casein has undergone the greatest ratio indicating the action of positive selection on this protein in the mouse lineage. This also happens to correspond to the lineage undergoing the highest shift in *pI *(Figure 1, Figure 2). This positive selection seems to have consequently shifted the *pI *of mouse κ-casein. Two other orthologs seem to have also undergone some sort of fast evolutionary divergence (Figure 2 shows that horse and cow have *dN/dS >*1) even though the *dN/dS *value might be too week to speak about positive selection, the cow ortholog happens to have also diverged in its *pI *(Figure 1 and Figure 2, horse however seems to have diverged in sequence but retained a closer basic *pI *to the other mammals studied in this work.).

For muc1, we detected 25 sites under positive selection (p < = 4.7.10^-2^), 15 of these have changed the overall *pI *of the protein and also changed its net charge. Here again, we find that there are significantly more sites undergoing positive selection and that these have an impact on the net charge of the protein compared to all other neutral sites (p = 2.4 × 10^-9^; 28% for charged residues versus 1.9% for neutral residues). Under a random distribution of the positively selected sites detected in the human muc1 protein sequence, we will expect an average of 4.4% sites that undergo positive selection whether these are charged or neutral, which is less than the observed 28% charged sites that have undergone positive selection.

Put together these results show that selection has played a part in the change of *pI *and consequently on the overall net charge of the protein.

### *Selection pressures for changes in pI*: *the roles of dietary, morphological, and intrinsic milk protein factors*

What is driving this selection on the *pI*? Can it be the important differences in *pH *and compartmentalization between the digestive systems of different mammals? [8] Milk proteins travel down the digestive system. Some, such as the caseins, get broken down in the highly acidic conditions of the stomach, whereas others such as lactadherin and lactoferrin [9,10] travel intact or partially intact to be broken down further down in the digestive tract. Given the very large shifts in *pI*, we would anticipate that the processing and breakdown of milk proteins are likely to differ substantially. Thus, if we were to replace the human κ-casein with that of mouse, it seems unlikely that they will interact with their environment and function in an identical way, given that the mouse and human κ-casein *pI *is 4.75 in mouse, but 8.59 in human.

We might imagine that the greatest shifts during evolution might occur when animals shift between largely carnivorous or omnivore diets and herbivore diets, since the more complex stomachs of some herbivores, and the more acid stomach *pH*s of some carnivores might alter functional constraints. However, inspection of Figure 1 indicates that many large shifts occur between species that have largely similar overall dietary strategies (dog and cat; mouse and rat). This suggests that the shifts in functional constraints may be associated with factors that are not linked with the gross morphology or diet of major clades. Similarly, the values of the posterior stomach *pH *in the different mammals represented in Figure 2 do not clearly argue for a stomach-*pH *change that is driving the shift in *pI *for κ-casein, including the significant *pI *shift observed in mouse (Fig1, Fig2, and Fig3). Besides, the great difference observed between the *pI *values of the two extra copies of κ-casein in platypus (Figure 1; *pI *= 5.9 for FJ548612, to *pI *= 8.8 for FJ548626) does not argue for a stomach *pH *driven selection on milk proteins' *pI*.

It is interesting to speculate on how extrinsic factors, such as commensal and pathogenic bacteria, may exert selection pressures on milk protein function, but also of interest to consider how alterations in intrinsic milk protein functions may relate to adaptive changes. Milk proteins are known to yield many bioactive peptides that modulate and participate in various regulatory processes in the body [11]. These peptides are usually cleaved by digestive enzymes such as trypsin, pepsin, and chymotrypsin. Some proteases cleave near positively charged residues, such as trpysin, while others avoid positive charge in their substrate region (pepsin), and the adaptive requirements for the gain and loss of proteolytic cleavage sites in certain regions of the gut (e.g. the duodenum versus the stomach) may have some an impact on *pI*. In particular, when we consider the casoxins [12], known bioactive peptides released from bovine κ-casein that have opioid antagonist and anti-opioid activities- we note that although casoxin A, and C are released in cow, this is not the case in human and mouse, since the cleavage sites are not the same between the species (Figure 4). It is interesting to note that 3 residues of the 14 residues that we found to be positively selected on in κ-casein are found on the borders of the three peptides casoxin A, B, and C (Figure 4), indicating possible selection on the cleavage sites. Also, Figure 4 shows that 3 other positively selected sites are located within the peptides casoxin A, and B sequence, indicating adaptation of the individual peptides at least to cow. Thus, the shift in *pI *may be associated with divergence in functional requirements for either rates of digestion, or for functional components of the milk.

### Phosphorylation and glycosylation

We observe that all the proteins that have shifted dramatically are ones that also happen to be highly glycosylated and phosphorylated. Indeed the three proteins κ-casein, muc1, and lactadherin have more glycosylation sites than the other milk proteins with an average of 7 glycosylations in human (9 glycosylations in muc1, 7 in κ-casein, and 5 in lactadherin; these include referenced, probable, and potential sites), and in cow, as opposed to an average of 1.3 in the remaining 6 milk proteins in human, and in cow. Besides, we do also observed differences in phosphorylation sites, for example we have 3 referenced phosphorylations in cow κ-casein and none in human and mouse. Also, there are 9 referenced phosphorylations in human muc1, while there are 6 and 7 by similarity in cow and mouse respectively.

Our analyses of *pI *did not take into account these post-translational modifications. To examine if post-translational modifications can reduce the difference in the isoelectric point, we used experimentally validated phosphorylation and glycosylation sites, which are defined in cow, human and to a weaker extent in mouse. For κ-casein (Figure 4), the cow *pI *shifts from 5.93 to 5.34 when the two experimentally verified phosphorylations are added. Human remains the same *pI *= 8.68 (no experimentally validated phosphorylation so far), and mouse shifts from 4.67 to 4.52 (1 potential phosphorylation site; Figure 4.). The phosphorylation sites for muc1 in both cow and mouse are potential sites found with similarity rather than experimentally validated sites. These results show that despite shifting the *pI *of κ-casein and muc1 towards a more acidic *pH *as a result of phosphorylation in the three different species, the difference in *pI *remains very important between these two proteins.

The differences in glycosylation between human and cow for κ-casein might somewhat further reduce the *pI *shift between both these species. Indeed, in κ-casein (Figure 4) we have 7 glycosylations in human as opposed to 6 in cow (none have been experimentally validated so far in mouse). For muc1, experimental validation is only available for human that has 4 O-linked, and 5 N-linked glycosylations. These might also narrow down the gap in the muc1 *pI *between the different species. Nonetheless, both cases where the *pI *difference is reduced or not are interesting. Indeed if the *pI *difference is reduced and becomes very close between both species, this reflects that the protein has adapted its *pI *so that the final product with the different number of glycosylations and phosphorylations becomes the same. Indeed, if the *pI *was initially not different, the addition of glycosylation will then further the gap between the *pI*s.

## Conclusions

Although the production of milk is conserved between mammals for over 190 MA, our results argue that common proteins that have been shared by mammals are functionally diverging. Many humans consume cow's milk on a daily basis, and yet the *pI *of κ-casein in cow is very different from our κ-casein. We have shown that selection has acted on the residues that affect the protein's *pI*. The simplest explanation was the adaptation of the protein to the different digestive systems to accommodate reactions to changes in *pH *of the different compartments. However, we found the pattern of change did not correlate strongly with the greatest shifts in compartmentalization and *pH *during evolution, suggesting that other factors, potentially including milk proteins' functional features, may be associated with the adaptive changes.

Differences in the function of κ-casein between various species, raises the question of whether κ-casein of cow can functionally replace that of human. κ-casein is known to yield many bioactive peptides [12,13] which, as we have discussed, might have different affinities and functionalities between human and cow. Such functional changes may relate to regional positive selection seen within κ-casein in the family bovidae [14].

It is of interest to note that two of the proteins showing the most striking shifts in *pI *are also glycosylated extensively (κ-casein and muc1). It is not clear if this is merely coincidental, or whether glycosylated proteins play a particular role in the gut that is subjected to shifting selection pressures over evolutionary time. An obvious candidate function would be bacterial interactions, which are heavily influenced by glycosylated proteins, and κ-casein is known to play a role in altering *Helicobacter pylori *adhesion [15] (review [16]). Exactly how shifting the *pI *of these milk proteins might benefit the neonate is not entirely clear. However, given the ability of pathogens such as *H. pylori *to modify the host stomach *pH *[17], the ability of milk proteins to coat particular compartments or infected regions of altered *pH *is an obvious candidate factor to investigate. In this context, a specific question raised by our study is whether the muc1 and κ-casein in cow's milk provide optimal protection against bacterial infections of the stomach and intestine for human neonates.

## Methods

### Data

The human, chimp, monkey macaque, mouse, rat, guinea pig, rabbit, cat, dog, horse, cow, opossum, and platypus protein sequences were downloaded by FTP from the ENSEMBL database at: ftp://ftp.ensembl.org/pub/release-63/fasta/

Out of seventeen identified major milk proteins  [18] we picked a subset for analysis on the basis of their belonging to at least 8 mammalian species out of the 13 (Table 2). In addition the 8 species needed to include human, chimp, cow, and mouse. These proteins represent the three parts of milk (Table 1): whey, casein, and milk fat globule. We used the 9 major milk proteins defined in human and cow to detect their orthologs in the 13 other genomes, defined by reciprocal hits.

### Orthologs and sequence evolution

To find orthologous non-milk proteins, we identified 13-way mutual best BLASTP hits among human, chimp, monkey macaque, mouse, rat, guinea pig, rabbit, cat, dog, horse, cow, opossum, and platypus. This method resulted in 1412 sets of putative orthologs that were present among all 13 species. Each set of 13 proteins was aligned using ClustalW [19].

### Calculating the isoelectric point

We first cleaved off the signal peptide from each protein using a HMM search with SignalP-HMM [20]. The rest of the sequence was incorporated into an in-house perl script for the calculation of the *pI *that uses the Henderson-Hasselbach equation. The script searched for the number of R, K, Y, C, H, E, and D that are implicated in the *pI *of a protein. Each of the previous amino acids was assigned a pK_a _value, 12.48, 10.54, 10.46, 8.18, 6.04, 4.07, and 3.9 respectively, 8.0 for the N-terminus, and 3.1 for the C-terminus. The charge due to arginine for example is the product of the corresponding pKa with the number of instances or R in the sequence. We can then calculate an estimated charge for the protein at any particular *pH*. To determine the *pI *that is the *pH *value at which the estimated charge is zero, we estimated an initial *pH *at which the overall charge of the protein is positive and one where the charge is negative. We then used a bisection method to estimate to a 10^-2 ^precision the value that renders the overall charge null.

### Defining significant pI shifting proteins

A protein is considered as significantly shifting in, for example mouse, if the distance between its *pI *and that of its ortholog in human is higher than a threshold that is determined from the differences in *pI *of all orthologs between human and mouse (Additional File 1). Setting a threshold of *pI *between two species is somewhat arbitrary because the data does not follow a known distribution, for this reason we used a non-parametric formula to define the threshold of significance. This threshold is calculated using the median, and third quartile of the absolute shift in *pI *between orthologous proteins this is: threshold = 2 × (3^rd ^quartile - median).

### Ancestral Reconstruction and amino acid substitution rate

To reconstruct the ancestral sequences of the current κ-casein protein, we aligned the κ-casein orthologs in the 12 species represented in Figure 2 using T-coffee [21]; this step was followed by a maximum likelihood reconstruction using codeml from the paml package [22].

To calculate the amino acid substitution we gathered the DNA coding sequences of κ-casein proteins from the ENSEMBL database. We could not locate good quality sequences for guinea pig, cat, and dog. We aligned the other 9 κ-casein protein orthologs using T-coffee [21]. The DNA sequences were aligned based on the protein alignment. We implemented codeml [22] on the DNA alignment to calculate the synonymous *dS *and non-synonymous *dN *substitutions.

### Detecting selection in the charged residues

To examine if the significant variation between human, chimp, mouse, and cow, in amino acid composition is due to selection, we gathered the DNA coding sequences of all milk-specific proteins from the ENSEMBL database. We aligned the proteins using T-coffee [21] and implemented a script that aligns to DNA based on the protein's alignment. We removed poorly aligned positions and divergent regions of a DNA alignment using Gblocks [23]. We used the SLR method with the default parameters to detect positions that are likely to be under positive selection [7]. These positions are indicated on Figure 4.

## Abbreviations

SLR: Sitewise Likehood Ratio.

## Competing interests

The authors declare that they have no competing interests.

## Authors' contributions

NK and DS conceived the study and wrote the paper. NK also carried out the analyses. Both authors read and approved the final manuscript.

## Supplementary Material

Additional file 1 **Table S1**. Threshold for large *pI *shifts between all the mammals and human. Each row contains the name of the species, the threshold above which a shift in *pI *is considered as important, and finally the number of proteins that satisfy the difference in *pI*. Click here for file

## Reviewers' comments

### Referee 1, Fyodor Kondrashov

#### Report form

This is a very interesting study of proteins present in milk. My only suggestion is to provide more background in the introduction section on the function of the proteins that were studied to give a reader that may not be well oriented in protein function a better understanding of the implications of their evolution.

#### Author's response

We have added a table (Table 1) summarizing the functions of the milk proteins used in this study. We refer to this table in different parts of the text including the introduction.

### Referee 2, David Liberles

#### Report form

"Shift in the isoelectric-point of milk proteins as a consequence of adaptive divergence between the milks of mammalian species" by Khaldi and Shields is an interesting paper examining functional shifts in mammalian milk proteins. An additional table and additional analyses are suggested to maximize the ability to interpret the data presented in the study.

**1) **First, a table with more detail on the known functions of milk proteins would be desirable and help the reader assess other adaptive processes beyond adaptation to digestive system pH.

#### Author's response

We have added a table (Table 1) summarizing the functions of the milk proteins used in this study. We refer to this table in different parts of the text including the introduction.

**2) **Additionally, a species tree showing known mammalian digestive system pH values at the tips coupled to reconstruction as continuous data over the tree would be informative. This couples to two additional pieces of information from the sequences. The first is a free ratios model of dN/dS (where supported) over each gene tree. The other is ancestral sequence reconstruction of sequences at nodes in trees and calculation of pI values for those ancestral states. These pieces of information can be used to ask if positive selection correlates with lineages where pI is changing and if this correlates with changes in digestive system pH values. Positive selection that is not explained by pI and pH changes would be strong candidates for alternative sources of adaptation.

With this, the authors will have performed a nice study of the evolution of milk proteins in mammals.

#### Author's response

We have added a figure (Figure 2) representing the ancestral reconstruction of κ-casein and the summary of the *pI *values and the *dN/dS *ratios (when possible) for each of the studied species (Figure 2). Figure 2 also shows most of the *pH *interval values of the anterior and posterior stomachs for the different mammals of this study. The results of Figure 2 are discussed in the results and discussion section.

#### Referee 3

This reviewer provided no comments for publication.
