# Supplementary material for: Shift in the isoelectric-point of milk proteins as a consequence of adaptive divergence between the milks of mammalian species
Source: Biol Direct. 2011 Jul 29;6:40. doi: 10.1186/1745-6150-6-40 (PMC3189186; doi:10.1186/1745-6150-6-40)
Supplement: Additional file 1 — Table S1. Threshold for large pI shifts between all the mammals and human. Each row contains the name of the species, the threshold above which a shift in pI is considered as important, and finally the number of proteins that satisfy the difference in pI. [file 1745-6150-6-40-S1.DOC]

**Additional Table 1**. Threshold for large *pI* shifts between all the mammals and human. Each row contains the name of the species, the threshold above which a shift in *pI* is considered as important, and finally the number of proteins that satisfy the difference in *pI*.

| Species | *pI* difference from human | Number of proteins |
| --- | --- | --- |
| chimp | 0.381 | 120 |
| Monkey macaque | 0.6066 | 178 |
| mouse | 0.92286 | 175 |
| rat | 0.96972 | 199 |
| guinea pig | 1.05468 | 137 |
| rabbit | 1.5501 | 160 |
| cat | 0.92286 | 179 |
| dog | 0.98145 | 152 |
| horse | 0.91551 | 182 |
| cow | 0.92286 | 167 |
| opossum | 1.23192 | 169 |
| platypus | 1.8384 | 158 |
